# Systematic review of clinical prediction models for psychosis in individuals meeting At Risk Mental State criteria

**DOI:** 10.3389/fpsyt.2024.1408738

**Published:** 2024-10-02

**Authors:** Alexandra Hunt, Heather Law, Rebekah Carney, Rachel Mulholland, Allan Flores, Catrin Tudur Smith, Filippo Varese, Sophie Parker, Alison R. Yung, Laura J. Bonnett

**Affiliations:** ^1^ Department of Health Data Science, University of Liverpool, Liverpool, United Kingdom; ^2^ Greater Manchester Mental Health NHS Foundation Trust, Manchester, United Kingdom; ^3^ School of Health Sciences, Division of Psychology & Mental Health, University of Manchester, Manchester, United Kingdom; ^4^ Institute for Mental and Physical Health and Clinical Translation (IMPACT), Deakin University, Geelong, VIC, Australia

**Keywords:** prognostic/prediction modelling, mental health, ARMS, psychosis, systematic review

## Abstract

**Objectives:**

This study aims to review studies developing or validating a prediction model for transition to psychosis in individuals meeting At Risk Mental State (ARMS) criteria focussing on predictors that can be obtained as part of standard clinical practice. Prediction of transition is crucial to facilitating identification of patients who would benefit from cognitive behavioural therapy and, conversely, those that would benefit from less costly and less-intensive regular mental state monitoring. The review aims to determine whether prediction models rated as low risk of bias exist and, if not, what further research is needed within the field.

**Design:**

Bibliographic databases (PsycINFO, Medline, EMBASE, CINAHL) were searched using index terms relating to the clinical field and prognosis from 1994, the initial year of the first prospective study using ARMS criteria, to July 2024. Screening of titles, abstracts, and subsequently full texts was conducted by two reviewers independently using predefined criteria. Study quality was assessed using the Prediction model Risk Of Bias ASessment Tool (PROBAST).

**Setting:**

Studies in any setting were included.

**Primary and secondary outcome measures:**

The primary outcome for the review was the identification of prediction models considering transition risk and a summary of their risk of bias.

**Results:**

Forty-eight unique prediction models considering risk of transition to psychosis were identified. Variables found to be consistently important when predicting transition were age, gender, global functioning score, trait vulnerability, and unusual thought content. PROBAST criteria categorised four unique prediction models as having an overall low-risk bias. Other studies were insufficiently powered for the number of candidate predictors or lacking enough information to draw a conclusion regarding risk of bias.

**Conclusions:**

Two of the 48 identified prediction models were developed using current best practice statistical methodology, validated their model in independent data, and presented low risk of bias overall in line with the PROBAST guidelines. Any new prediction model built to evaluate the risk of transition to psychosis in people meeting ARMS criteria should be informed by the latest statistical methodology and adhere to the TRIPOD reporting guidelines to ensure that clinical practice is informed by the best possible evidence. External validation of such models should be carefully planned particularly considering generalisation across different countries.

**Systematic review registration:**

https://www.crd.york.ac.uk/PROSPEROFILES/108488_PROTOCOL_20191127.pdf, identifier CRD42018108488.

## Introduction

Identification of individuals at high and imminent risk of developing a first episode of psychosis (FEP) is possible through the use of the “At Risk Mental State” (ARMS) criteria (also known as the Ultra High Risk (UHR) and Clinical High Risk (CHR) criteria) (1, 2). These criteria were originally proposed by Yung and are operationally defined as low-grade “psychotic-like symptoms” that cause distress (3). Meta-analytic evidence indicates that about 15%–22% of ARMS individuals develop psychosis within 12 months from ARMS assessment (4, 5). Identification of ARMS individuals therefore presents the opportunity for early intervention to prevent the onset of psychosis. However, most individuals meeting the ARMS criteria will not develop psychosis. This means that some ARMS individuals might be receiving unnecessary treatment and that the health services may be using costly interventions (preventive interventions with a growing evidence-based, e.g., cognitive behavioural therapy, CBT) in people who may not need it. Furthermore, there are growing calls to improve the routine clinical management of people with ARMS. For example, the United Kingdom’s Early Intervention Access and Waiting Standards now require all early intervention in psychosis services to assess, monitor, and manage ARMS individuals (6). While this will benefit individuals who will be afforded more timely care, it is likely to result in an additional strain on resources. There is an urgent need to develop better systems to identify ARMS individuals that might be at the highest risk of developing psychosis and who might therefore particularly benefit from receiving evidence-based preventive interventions.

Alternative predictors for assessing risk of transitioning to psychosis do exist, such as basic symptoms (7). Prediction of risk in this way is comparable or, in some cases, slightly superior than predicting transition to psychosis using ARMS (4). However, ARMS as assessed by the Comprehensive Assessment of At Risk Mental States (CAARMS) is the most widely used approach to define risk for psychosis worldwide and forms part of national clinical assessment in the United Kingdom (6). Indeed, ARMS is a standard approach within the United Kingdom, with the whole workforce being trained in assessment and detection using the CAARMS. Consequently, there are specific calls around more effective prediction of FEP in those meeting the ARMS criteria as defined by the CAARMS (4, 8).

Prognostic factors identify groups of patients at highest risk and thus inform treatment decision making, patient counselling, and policies. Clinical prediction models combine multiple prognostic factors to predict individual outcome risk for individuals (9). Research to better stratify ARMS patients according to levels of risk of psychosis could facilitate more efficient use of resources available to health services. For example, those predicted to be at highest risk could be offered CBT, while lower risk patients could be offered less costly and less-intensive regular mental state monitoring. Potential predictors that can be easily measured in routine clinical practice are the most relevant as they can be used to create a practical tool for risk prediction. Clinical and cognitive tests are less burdensome to patients and more feasible for staff to administer than electrophysiological, imaging, and other non-standard tests and have at least equivalent predictive accuracy (10, 11). We, therefore, restricted our review to predictors that can be obtained as part of standard clinical practice. Application of a clinical prediction tool that can be used in routine practice could lead to effective identification of individuals at risk of psychosis, the development of more cost-effective pathways and management plans.

Two recent systematic reviews summarised existing prediction models for ARMS patients (12, 13). Both systematic reviews were undertaken in 2017, and since then, many additional relevant prediction models have been published.

## Aims of the review

The aim of this systematic review was to identify and summarise clinical prediction models predicting transition of people meeting the ARMS to FEP at 12 months, irrespective of whether an individual received an intervention or not. Our review focussed on predictors that can be obtained as part of standard clinical practice rather than those requiring additional procedures or assessments. In summarising models, the review also evaluated the risk of bias introduced and the areas that are common pitfalls in prediction modelling.

## Methods

This systematic review was conducted according to Preferred Reporting Items for Systematic Reviews and Meta-Analyses (PRISMA) guidelines for reporting systematic reviews (14). A protocol for the review was registered with PROSPERO (CRD42018108488) and published in Diagnostic and Prognostic Research (15).

### Search strategy

The following bibliographic databases were searched: PsycINFO, Medline, EMBASE, and CINAHL, all from January 1994 to 8 July 2024. 1994 was selected as the start date, as this was the initial year of the first prospective study using ARMS criteria (16). Searches used index terms and text words that encompassed the patient group supplemented by terms relating to transition and prognostic factors (see sample Medline search in **Appendix 1**). No language restrictions were placed on the searches.

The search strategy was developed according to published guidelines for the identification of prediction models (17). The strategy for the identification of people with ARMS was written by a subject information specialist with the University of Liverpool.

Database searches were supplemented by inspection of studies included in previous systematic reviews and meta-analyses of psychosis transition studies, reference lists of psychosis transition studies identified through the database searches, and citations of psychosis transition studies identified through the database searches.

### Selection/inclusion criteria

#### Study design

The review included prospective or retrospective studies (i.e., cohort studies and randomised controlled trials of preventive interventions), with participants meeting the ARMS criteria that developed, compared, or validated a prediction model or clinical prediction rule based on a model, combining multiple predictors to predict the risk of transition to psychosis.

#### Population

Individuals meeting ARMS criteria [also called Ultra High Risk (UHR) or Clinical High Risk (CHR) criteria]. These are defined as i.e. 1) attenuated psychotic symptoms, 2) full blown intermittent psychotic symptoms and 3) genetic/familial risk for schizophrenia in conjunction with a significant decrease in functioning and operationalised using suitable measures such as the Comprehensive Assessment of At-Risk Mental States (CAARMS) (18) or the Structured Interview for Prodromal Syndromes (SIPS) (19). Other prodromal signs/symptoms distinct from ARMS (e.g., basic symptoms) were not included. Studies with mixed populations, including those outside of the remit, were included provided that the appropriate data for our defined group of patients were extractable.

#### Intervention (potential prediction models)

Studies must have reported a clinical prediction model including multiple variables to predict the risk of transition to psychosis following confirmation that a patient meets the ARMS criteria. A prediction model was defined as a combination of at least two patient characteristics (multivariable) within a statistical model (e.g., a regression model or, increasingly more commonly, machine learning) to predict an individual’s risk, or probability, of transition (20, 21). Our review focussed on predictors that can be obtained as part of standard clinical practice rather than those requiring additional procedures or assessments.

#### Control

There is no comparator for this review, as the existence of alternative models was not considered in our review.

#### Outcome

Eligible prediction models included patients at risk of transitioning to psychosis and thus recruited to the study at the time of the ARMS assessment. The primary outcome was to identify prediction models that were assessed as having a low risk of bias, in line with the PROBAST guidelines, when considering an individual’s risk of experiencing FEP. This is defined using standard diagnostic classification systems (DSM-III, DSM-IV, DSM-5, ICD-10, ICD-11) or commonly used ARMS assessment schedules (e.g., CAARMS or SIPS). This outcome was considered while assessing the predictive quality of the developed models in terms of use of appropriate statistical methodology, and the feasibility of using the model in clinical practice.

The secondary outcome was to identify predictors frequently used within identified prediction models and make use of frequently occurring predictors for further research.

#### Timing

The primary outcome was FEP within 12 months of an ARMS assessment.

#### Setting

Studies in any setting were included.

### Study selection

Study selection followed a two-step process. Titles and abstracts were initially screened for relevance by two reviewers independently using pre-defined screening criteria. The screening criteria were broad and considered whether studies included patients meeting the ARMS criteria and developed or examined prediction models in relation to transition to psychosis.

Full texts of any potentially relevant articles were then obtained, and two reviewers independently assessed the studies against the full inclusion criteria. Any discrepancies between reviewers were resolved by discussion or referral to a third reviewer. Portions of non-English language studies were translated where necessary to facilitate study selection to subsequent data extraction. The study selection process was documented using the PRISMA flow diagram (22, 23). EndNote reference management software was used to remove duplicates (24), while Microsoft Excel workbook was used to record reviewer decisions, including reasons for exclusion (25).

### Data extraction

Data extraction from included studies was conducted independently using an in-depth piloted data extraction form. Disagreements were resolved through discussion or referral to a third reviewer. Data extraction considered study characteristics, study design characteristics, patient characteristics, candidate prognostic factors considered including information on missing data, outcome measures, statistical methods employed and how prognostic factors included in the analysis were handled, and prediction model information. The measurement method of prognostic factors and the applicability of these for future prediction models were considered. Our review focussed on predictors that can be obtained as part of standard clinical practice rather than those requiring additional procedures or assessments. Data extraction specifically relating to the clinical prediction models assessed as a low risk of bias included any internal and/or external validation performance statistics for discrimination (such as the c-statistics or area under the curve) or for calibration (such as the expected/observed events ratio).

### Assessment of study quality

The risk of bias (quality) of included studies was assessed using the criteria described by Altman (26) and by the Prediction model Risk Of Bias ASessment Tool (PROBAST) (27). PROBAST involves the assessment of participants, predictors, outcomes, and analysis.

### Summarising identified evidence

Any studies reporting the development of a prediction model were summarised narratively, in particular what prognostic factors were included in the final model and whether the model was validated internally and/or externally. Information was tabulated and plots or visual representations were created when felt necessary. The PROBAST evaluation was used to determine the risk of bias of the model (i.e., whether the model is likely to work as intended for the ARMS population of interest), with models classed as low, unclear, or high risk of bias.

## Results

### Studies identified

**Figure 1:**
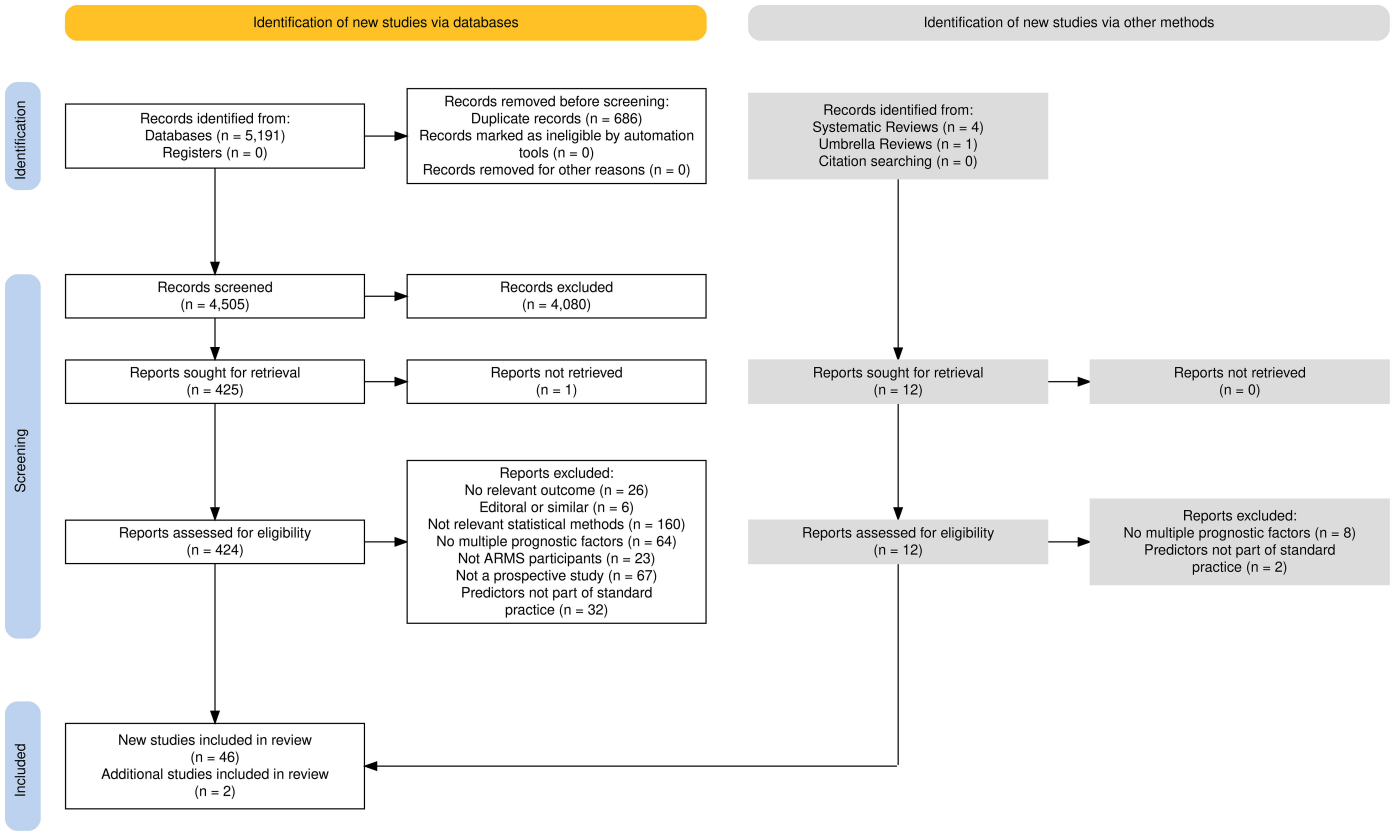
PRISMA flow diagram.

A PRISMA flow diagram, shown in **Figure 1**, presents the studies included within this systematic review.

The search yielded 5,191 results across the four databases, 686 of which were excluded as they were duplicate studies. The remaining titles and abstracts were screened to obtain 425 results, whose titles and abstracts were deemed to be relevant according to the pre-defined criteria. Most studies were published in English although the title and abstract of 11 studies were translated into English: five French, three German, two Japanese, and one Swedish studies. These studies were excluded after screening for title and abstracts, and no further translations were required.

Using pre-defined selection criteria (see **Table 1**) applied to full texts, 347 studies were excluded. Reasons for exclusion included no development of a prediction model (*n* = 160), univariable analysis rather than multivariable model building (*n* = 64), no transition to psychosis as an outcome (*n* =26), incorrect patient group (no ARMS individuals) (*n* = 23), editorial rather than full report (*n* = 5), and study design other than those listed in our inclusion criteria (*n* = 66). One study’s full text was not accessible. This left 78 studies for inclusion in the review. Thirty-two studies included clinically assessed predictors, such as brain scans or blood tests. Predictors such as these would require a lengthy in-depth clinical assessment within a hospital, which was beyond the scope of this review, as the priority is a model that can be applied within standard clinical practice. Of the 78 identified studies, 32 studies included neurocognitive predictors that would need to be assessed in clinic. Therefore, the remaining 46 studies with predictors easily obtained within standard clinical practice, such as age, gender, or even predictors that required an assessed interview, were assessed for this review.

**Table 1:** Inclusion and exclusion criterion.

Inclusion criteria		Exclusion criteria	
1	Studies relevant to psychosis and mental health	1	Studies without the relevant outcome, transition to psychosis
2	Populations including participants presenting At Risk Mental State (ARMS) commonly assessed by CAARMS or SIPS. Mixed populations were included, as long as ARMS individuals existed in the population.	2	Studies not presenting prediction modelling methods.
3	Publications dated from 1994 to July 2024.	3	Studies not presenting ARMS participants.
4	Retrospective and prospective study designs. (e.g., cohorts, observational, registry data).	4	Reviews (studies not presenting original data).
5	Standard predictors that can be obtained as part of routine standard clinical practice.		

The 185 identified systematic reviews were then assessed to determine any additional models to be included in the review. Four systematic reviews (10, 12, 13, 28) and one umbrella review were found to be of relevance (29). From these, two additional prediction models of relevance to this review were identified, thus making the total number of models under review 48.

The summary of each study and a complete list of all included studies, including appropriate references, can be found in **Supplementary Tables S1**, **S2**, respectively. The age of participants within the including studies ranged from 12 to 40 years old (*M* = 20.81)

### PROBAST quality assessment

The full PROBAST assessment for included studies can be found in **Supplementary Table S3**. Overall, four studies were graded as low risk of bias and 38 studies as high risk of bias (**Table 2**). Justification of these classifications and the applicability of the models within clinical practice follows.

**Table 2:** Risk of bias of included studies across the four PROBAST domains.

Level of bias	Participant selection	Predictor	Outcome	Analysis	Overall
High risk of bias	4	17	15	31	**40**
Low risk of bias	42	22	12	6	**4**
Moderate/unclear risk of bias	2	9	21	11	**4**

### Included predictors

Among the 48 studies included, 103 different predictors were identified, and 42 of these were used more than once within different studies. See **Supplementary Table S4** for all predictors identified. **Table 3** displays the top ten frequently occurring predictors identified within the review.

**Table 3:** Top 10 frequently occurring predictors identified within the 46 identified studies.

Predictors (top 10)	Frequency (%)
Global Assessment Functioning (GAF) Score	17 (17.4)
Age	14 (14.3)
Trait group (vulnerability)	11 (11.2)
Unusual thought content (UTC)	9 (9.2)
Gender	8 (8.2)
Positive symptoms	8 (8.2)
Social and Occupational Functioning Assessment Score	7 (7.1)
Decline in GAF	7 (7.1)
Sum of SIPS (UTC + suspiciousness)	7 (7.1)
Brief Assessment of Cognition in Schizophrenia	6 (6.1)

#### Risk of bias

##### Participants

Data from observational studies were used to develop prediction models within all studies. Observational studies are most informative in prognostic studies, and thus these studies have been graded as low risk of bias (*n* = 48) (30).

Forty-two (87.5%) studies provided reasons for exclusion and inclusion of participants and were graded as low risk of bias. The two most popular exclusion criteria were low IQ (< 85) (*n* = 21) and past or present psychotic disorders (*n* = 20). It was necessary to grade two studies as unclear and four as high risk due to unrepresentative inclusion criteria such as a criterion of exclusively right-handed Caucasian individuals or the inclusion of self-referred individuals.

##### Predictors

In order to reduce bias that is introduced during selection of participants, model predictors must be defined in the same way for each participant. We did not identify any studies that defined predictors differently for participants. Therefore, all studies were graded as a low risk of bias among the definition of predictors.

##### Outcome

Thirty-eight (79.2%) included studies used pre-specified outcomes although often the predictors were included in the outcome. Therefore, overall, we deemed there to be a moderate to high risk of bias from participant selection in most studies.

Thirteen studies demonstrated a low risk of bias. These studies identified their pre-specified outcome of transition to psychosis alongside references to literature definitions, with clinical knowledge or guidance. It is important that these studies used the same thresholds and categories to define the presence of the outcome and a similar interval between predictor assessment and outcome determination. These 13 studies all presented a follow-up > 1 year.

##### Analysis

Sample size varied from *n* = 34 to *n* = 1,676, and the number of events per variable (EPV = number of transitions to psychosis divided by the number of levels of all candidate predictors) varied from 1.846 to 48.25. This ratio was not estimable in eight studies, which did not report either the number of transitions (*n* = 8) or/and did not specify a list of candidate predictors (*n* = 2). This was usually in studies that reported results from multiple model variations. Commonly cited evidence for a minimum number of events per variable is 10 (31, 32), although some evidence suggest 50 might be more appropriate (33). This has now been replaced by more study-specific calculations of event numbers (34). The presence of missing predictor data will further reduce the apparent EPV (see **Supplementary Figure S5**). Only eight studies achieved at least 10 events per variable; however two of these based their choice of final model predictors on univariable analyses, which is not a recommended statistical practice. Another two studies made use of the Hosmer–Lemeshow goodness-of-fit test to assess the calibration of the predictive logistic regression model. The Hosmer–Lemeshow goodness-of-fit test is not a currently recommended practice (35); therefore, both were graded as a moderate risk of bias. The remaining 43 studies introduced bias.

Continuous predictors were occasionally categorised (*n* = 3). Univariable model selection was employed (*n* = 8), and the methodology behind the multivariable model building is usually not described. One study opted for forward selection rather than the statistically preferred backwards selection (30).

Ten of the studies had participants excluded from the final analysis and did not account for missing data and thus were graded as high risk of bias. Most studies (*n* = 29, 60.4%) provided no performance measures for the developed model. Of those that did, sensitivity and specificity were the most commonly reported performance measures (*n* = 23). Twenty-five studies included a form of internal validation, while eight included external validation of the developed model. Of these, one study either recalibrated or showed evidence that no recalibration was needed through a calibration plot. Methodology for internal validation included the statistically preferred bootstrap resampling in 15 studies and the less favoured cross-validation in two studies (36). Bootstrapping varied from 200 iterations to 5,000. Two studies used multiple imputation to counteract extensive missing data.

Risk of bias based on analysis was therefore considered to be high in 30 studies, moderate in 12, and low in 6.

##### Overall

Considering the four risks of bias domains (see **Table 2**), in total, forty (83.3%) studies were rated as high risk of bias overall, four as medium, and four as low. **Supplementary Table S6** presents the predictors used within the four studies graded as low risk of bias.

Of the four low-risk studies, three studies presented C-statistics based on development data and two studies showed C-statistic based on bootstrap internal validation. One of the four studies presented a value for calibration slope. Values for discrimination and calibration can be seen in **Supplementary Table S7.**

#### Applicability

##### Participants

The ARMS assessment has a strict criterion for an individual to meet ARMS; this was within our inclusion criteria for the studies. Some studies had a mixed population of ARMS individuals and others. In a limited number of studies (*n* = 8), the group of participants included control groups and individuals with other mental health issues, whereby the data solely for ARMS individuals could not be isolated. The remaining 40 studies have a low concern regarding applicability of participants.

##### Predictors

Studies considering predictors which can be assessed, and defined in the same way for all participants and were blinded or masked to the outcome data, have been graded as low risk of concern (*n* = 22). Those requiring assessments with no indication of blinding have been graded as medium risk of concern (*n* = 9). Those with missing predictor data at the time of building or validating the model and/or included unstable predictors due to lack of blinding between the predictor and the outcome data (*n* = 17) were graded as high risk of concern regarding applicability bias.

##### Outcome

Transition to psychosis is a recognised and clearly defined outcome within mental health research. Therefore, there are no concerns regarding applicability with any of the included studies. Eleven studies failed to define the outcome, but given the widely accepted definition of psychosis, we have only downgraded these studies to moderate concern of applicability. Twenty-eight studies demonstrated that one or more predictors formed part of the definition or assessment of outcome, known as incorporation bias, and/or the outcome determination varied among participants. These were graded as a high risk of bias.

Seven studies defined a secondary outcome, such as a second psychotic episode after another specified follow-up period (*n* = 3), illness severity/level of functioning (*n* = 3), and effects of medication or treatment (*n* = 2).

##### Overall

Considering the four risks of bias domains for applicability, in total, forty studies were rated as high risk of bias overall, four as medium, and four as low.

## Discussion

This systematic review provides a summary of existing clinical prediction models predicting transition to FEP at 12 months in individuals meeting ARMS criteria. This review has been conducted to a high standard focussing on assessing the quality and risk of bias within studies identified for considering transition risk in ARMS participants and identified 103 different predictors used across the included 48 studies. The top 3 most frequently occurring predictors were Global Assessment of Functioning (GAF) score, age, and trait group (genetic vulnerability). Four studies were assessed as low risk of bias, thirty-eight as high risk of bias, and four as moderate or unclear risk of bias overall.

Many prediction models are developed each year, and previous systematic reviews have evaluated the methodological conduct and reporting of studies developing prediction models (37–39). Many conclude that model development studies are characterised by deficiencies in study design, inadequate statistical methodology, and poor reporting. Since the publication of previous relevant reviews in 2017, our review suggests that there has been little improvement in methodology of model development or the reporting of model development in this field.

We critically appraised the quality of 48 prediction model studies for transition to psychosis in people meeting ARMS criteria using the PROBAST tool (40). The review did not exclude non-English studies, which are not common of other systematic reviews; therefore, this review contains cross-language research and thus reduces bias introduced through language barriers. It has been noted that previous reviews did not identify any external validation studies; however, our review identified eight such studies.

Our review is the latest systematic review in the field and thus includes recently published prediction models as well as historic ones. We have also included models that have used any statistically appropriate method to consider the outcome. This includes logistic regression, which was not included in Sanfelici’s systematic review (10). Also, unlike the existing published systematic reviews in the field, ours includes a comprehensive assessment of risk of bias via the PROBAST checklist (27). PROBAST was developed by a steering group that considered existing ROB tools and reporting guidelines. The tool was informed by a Delphi procedure involving 38 experts and was refined through piloting and enables a focussed and transparent approach to assessing the ROB and applicability of studies that develop, validate, or update prediction models for individualised predictions (40). Its use is now strongly encouraged within any systematic review of prediction models and thus our review is conforming to the latest statistical standards (30).

As with any research project, there are limitations. For example, our systematic review consisted of studies with participants or subgroups treated with medication or common anti-psychotic therapies, such as CBT (41). Although we made use of this within our PROBAST review, the differences regarding the administration of medications, duration of treatment and different standard protocols, and guidelines across studies may have introduced cofounding bias. Heterogeneity of studies also makes it difficult to compare directly the different ways of classifying ARMS (e.g., inclusion of basic symptoms). Few studies meeting high quality and low risk of bias suggest a need to promote adoption of the TRIPOD reporting guidelines (21).

Clinical prediction models aid healthcare decisions in estimating the occurrence of a future event, usually in the presence of a disease or condition. But ample amounts of evidence reveal a poor quality of reporting of prediction model studies, be that model development or model validation. This means that clinical prediction models may be used inappropriately, introducing the possibility that some high-risk individuals are missed and some low-risk individuals are receiving unnecessary treatment. In 2015, the Transparent Reporting of a multivariable prediction model for Individual Prognosis Or Diagnosis (TRIPOD) guidelines were published and provide a checklist for researchers when publishing their work (21). The use of the TRIPOD reporting guidelines should significantly reduce the poor reporting and facilitate use of a more robust statistical methodology due to the inclusion of an associated elaboration document (21). However, submission of the TRIPOD checklist is yet to be compulsory when submitting a prediction model for publication. Therefore, work needs to be undertaken to encourage journal editors to make submission of a TRIPOD checklist compulsory akin to CONSORT checklists for randomised controlled trials (42).

Since April 2016, National Health Service (NHS) England has required all Early Intervention in Psychosis Services to assess and manage individuals with an ARMS. Research to better stratify ARMS patients according to levels of risk of psychosis is lacking. Prediction models have the potential to assist with stratification of risk. However, such models require development using statistically robust methodology, and validation both internally, and in independent data, which reflects the required generalisability (such as across countries). Our systematic review not only highlighted weaknesses within this area but also identified different variables available to test and consider within many areas of mental health, functioning and neurocognitive performances. This work is a crucial step towards the evidence-based use of prognostic factors for risk prediction in patients with ARMS considering treatment and leads to the development of new research ideas, which can have a profound impact on policy, commissioning, and patient care.

## Data Availability

The original contributions presented in the study are included in the article/**Supplementary Material**. Further inquiries can be directed to the corresponding author.

## Appendix 1

Medline search strategy:

1. (ARMS or At Risk Mental State).ti,ab.

2. Basic symptoms.ti,ab.

3. Prodromal psychosis.ti,ab.

4. Psychosis risk.ti,ab.

5. (UHR or Ultra High Risk or CHR or Clinical High Risk).ti,ab.

6. Prodrom*.ti,ab.

7. Psychosis*.ti,ab.

8. 6 and 7

9. 1 and 7

10. 5 and 7

11. 1 or 2 or 3 or 4 or 5 or 8 or 9 or 10

12. (transition or transition$).ti,ab.

13. exp “Predictive Value of Tests”/

14. predict$.ti,ab.

15. exp Risk/

16. risk$.ti,ab.

17. prognos$.ti,ab.

18. or/13-17

19. 11 and 12 and 18
