# Supplementary material for: Systematic review of clinical prediction models for psychosis in individuals meeting At Risk Mental State criteria
Source: Front Psychiatry. 2024 Oct 2;15:1408738. doi: 10.3389/fpsyt.2024.1408738 (PMC11480010; doi:10.3389/fpsyt.2024.1408738)
Supplement: Supplementary file 1 [file DataSheet1.pdf]

Supplementary Material

Supplementary Material Table S1: Summary of included studies

Study Reference	Year of publication	Country (of data/recruitment of individuals)	Age of participants (mean)	Age of participants (range)	Total Sample population	No. predictors within developed/validating model	Mixed Sample population?
Addington (1)	2016	Canada	-	-	179	7	no
Bang (2)	2015	South Korea	20.3	[13-35]	60	8	no
Bedi (3)	2015	USA	-	14-27	34	-	no
Bourgin (4)	2020	France	17.63	[15-25]	27	4	no
Brodey (5)	2019	USA	20.5	[18-35]	149	-	no
Cannon (6)	2016	USA	18.5	-	596	8	no
Cannon (7)	2008	North America	18.1	-	291	-	no
Carrion (8)	2016	USA	16.6	[12-25]	176	8	no
Chan (9)	2019	Singapore	20.8	[16-30]	343	10	no
Ciarleglio (10)	2018	USA	20.8	[13-30]	199	17	no
Conrad (11)	2017	Australia	17.5	-	191	14	yes
Dragt (12)	2011	Netherland	19	12-35	72	9	no
Formica (13)	2022	Melbourne	18.5	-	105	4	no
Francesconi (14)	2016	Italy	24.5	-	67	4	yes
Fusar-Poli (15)	2017	England	32.97	-	368	5	yes
Guo (16)	2020	USA	16.5	[12-25]	35	4	no
Haidl (17)	2018	"6 European countries"	23.7	[16-35]	230	5	no
Hengartner (18)	2017	Switzerland	20.5	[13-35]	188	13	no
Ising (19)	2016	The Netherlands	22.73	[14-35]	185	5	no
Kotlicka-Antczak (20)	2019	Poland	18.8	[16-20]	105	2	no
Koutsouleris (21)	2021	England, Finland, Germany, Italy, North America, Switzerland	21.23	-	930	6	no
Lee (22)	2022	South Korea	21.25	-	208	6	no
Lindgren (23)	2021	Finland	16.5	[15-18]	145	8	no

Malda (24)	2019	Worldwide	-	-	1676	8	no
Mechelli (25)	2016	Australia	-	[15-30]	416	-	no
Michel (26)	2014	Germany	24.7	[16-40]	97	5	no
Nelson (27)	2010	Australia	18.34	[15-30]	92	7	yes
Padmanabha (28)	2016	Pennsylvania, Finland	15.8	-	83	9	no
Paetzold (29)	2021	Australia, England, the Netherlands	23.6	[15-35]	48	2	no
Pawelczyk (30)	2021	Poland	19.15	[15-32]	73	3	no
Polari (31)	2021	Australia, Asia, Europe	19.1	[13-40]	202	3	yes
Riecher-Rossler (32)	2009	Switzerland	26.3	-	53	3	no
Ruhrmann (33)	2010	Germany, Finland, the Netherlands, England	23	[16-32]	245	6	no
Seidman (34)	2016	North America	-	[12-35]	689	4	no
Studerus (35)	2020	Switzerland	25	-	196	-	no
Tarbox (36)	2014	North America	19.7	-	54	-	no
Thompson (37)	2011	Australia	19.4	[14-30]	104	5	no
Uttinger (38)	2018	Switzerland	34.9	-	186	4	yes
Valmaggia (39)	2013	Australia, England	20.3	[14-35]	318	-	no
Worthington (40)	2020	USA	18.7	[12-35]	417	9	no
Yuen (41)	2019	Australia	18.4	-	382	-	no
Yuen (42)	2018	Australia, Austria, Demark, Japan, the Netherlands, Singapore, Switzerland	19.1	-	304	14	no
Yung (43)	2002	Australia	19.1	[14-28]	49	8	no
Yung (44)	2010	Germany, Finland, the Netherlands	-	[16-35]	245	6	no
Zhang (45)	2019	USA, China	19.5	-	196	4	no
Zhang (46)	2019	China	-	-	349	4	no
Zhang (47)	2020	China	-	[12-25]	199	7	yes
Zhang (48)	2018	China	19.1	-	199	6	no

Supplementary Material Table S2: List of 48 included studies

1. Addington J, Liu L, Perkins DO, Carrion RE, Keefe RS, Woods SW. The Role of Cognition and Social Functioning as Predictors in the Transition to Psychosis for Youth With Attenuated Psychotic Symptoms. Schizophr Bull. 2017;43(1):57-63.
2. Bang M, Kim KR, Song YY, Baek S, Lee E, An SK. Neurocognitive impairments in individuals at ultra-high risk for psychosis: who will really convert? Australian & New Zealand Journal of Psychiatry. 2015;49(5):462-70.
3. Bedi G, Carrillo F, Cecchi GA, Slezak DF, Sigman M, Mota NB, et al. Automated analysis of free speech predicts psychosis onset in high-risk youths. npj Schizophrenia. 2015;1(1):15030.
4. Bourgin J, Duchesnay E, Magaud E, Gaillard R, Kazes M, Krebs MO. Predicting the individual risk of psychosis conversion in at-risk mental state (ARMS): a multivariate model reveals the influence of nonpsychotic prodromal symptoms. Eur Child Adolesc Psychiatry. 2020;29(11):1525-35.
5. Brodey BB, Girgis RR, Favorov OV, Bearden CE, Woods SW, Addington J, et al. The Early Psychosis Screener for Internet (EPSI)-SR: Predicting 12 month psychotic conversion using machine learning. Schizophr Res. 2019;208:390-6.
6. Cannon TD, Yu C, Addington J, Bearden CE, Cadenhead KS, Cornblatt BA, et al. An individualized risk calculator for research in prodromal psychosis. American Journal of Psychiatry. 2016;173(10):980-8.
7. Cannon TD, Cadenhead K, Cornblatt B, Woods SW, Addington J, Walker E, et al. Prediction of psychosis in youth at high clinical risk: a multisite longitudinal study in North America. Arch Gen Psychiatry. 2008;65(1):28-37.
8. Carrión RE, Cornblatt BA, Burton CZ, Tso IF, Auther AM, Adelsheim S, et al. Personalized prediction of psychosis: external validation of the NAPLS-2 psychosis risk calculator with the EDIPPP project. American Journal of Psychiatry. 2016;173(10):989-96.
9. Chan CT, Abdin E, Subramaniam M, Tay SA, Lim LK, Verma S. Two-year clinical and functional outcomes of an Asian cohort at ultra-high risk of Psychosis. Frontiers in Psychiatry. 2019;9:758.
10. Ciarleglio AJ, Brucato G, Masucci MD, Altschuler R, Colibazzi T, Corcoran CM, et al. A predictive model for conversion to psychosis in clinical high-risk patients. Psychol Med. 2019;49(7):1128-37.
11. Conrad AM, Lewin TJ, Sly KA, Schall U, Halpin SA, Hunter M, et al. Utility of risk-status for predicting psychosis and related outcomes: evaluation of a 10-year cohort of presenters to a specialised early psychosis community mental health service. Psychiatry Res. 2017;247:336-44.
12. Dragt S, Nieman DH, Veltman D, Becker HE, van de Fliert R, de Haan L, et al. Environmental factors and social adjustment as predictors of a first psychosis in subjects at ultra high risk. Schizophrenia Research. 2011;125(1):69-76.
13. Formica MJC, Phillips LJ, Hartmann JA, Yung AR, Wood SJ, Lin A, et al. Has improved treatment contributed to the declining rate of transition to psychosis in ultra-high-risk cohorts? Schizophr Res. 2022;243:276-84.
14. Francesconi M, Minichino A, Carrión RE, Delle Chiaie R, Bevilacqua A, Parisi M, et al. Psychosis prediction in secondary mental health services. A broad, comprehensive approach to the "at risk mental state" syndrome. Eur Psychiatry. 2017;40:96-104.
15. Fusar-Poli P, Rutigliano G, Stahl D, Davies C, Bonoldi I, Reilly T, et al. Development and Validation of a Clinically Based Risk Calculator for the Transdiagnostic Prediction of Psychosis. JAMA Psychiatry. 2017;74(5):493-500.
16. Guo JY, Niendam TA, Auther AM, Carrión RE, Cornblatt BA, Ragland JD, et al. Predicting psychosis risk using a specific measure of cognitive control: a 12-month longitudinal study. Psychol Med. 2020;50(13):2230-9.

17. Haidl T, Rosen M, Schultze-Lutter F, Nieman D, Eggers S, Heinimaa M, et al. Expressed emotion as a predictor of the first psychotic episode — Results of the European prediction of psychosis study. Schizophrenia Research. 2018;199:346-52.
18. Hengartner MP, Heekeren K, Dvorsky D, Walitza S, Rössler W, Theodoridou A. Checking the predictive accuracy of basic symptoms against ultra high-risk criteria and testing of a multivariable prediction model: Evidence from a prospective three-year observational study of persons at clinical high-risk for psychosis. Eur Psychiatry. 2017;45:27-35.
19. Ising HK, Ruhrmann S, Burger NA, Rietdijk J, Dragt S, Klaassen RM, et al. Development of a stage-dependent prognostic model to predict psychosis in ultra-high-risk patients seeking treatment for co-morbid psychiatric disorders. Psychol Med. 2016;46(9):1839-51.
20. Kotlicka-Antczak M, Karbownik MS, Stawiski K, Pawełczyk A, Żurner N, Pawełczyk T, et al. Short clinically-based prediction model to forecast transition to psychosis in individuals at clinical high risk state. Eur Psychiatry. 2019;58:72-9.
21. Koutsouleris N, Worthington M, Dwyer DB, Kambeitz-Ilankovic L, Sanfelici R, Fusar-Poli P, et al. Toward Generalizable and Transdiagnostic Tools for Psychosis Prediction: An Independent Validation and Improvement of the NAPLS-2 Risk Calculator in the Multisite PRONIA Cohort. Biological Psychiatry. 2021;90(9):632-42.
22. Lee TY, Hwang WJ, Kim NS, Park I, Lho SK, Moon SY, et al. Prediction of psychosis: model development and internal validation of a personalized risk calculator. Psychol Med. 2022;52(13):2632-40.
23. Lindgren M, Kuvaja H, Jokela M, Therman S. Predictive validity of psychosis risk models when applied to adolescent psychiatric patients. Psychol Med. 2023;53(2):547-58.
24. Malda A, Boonstra N, Barf H, de Jong S, Aleman A, Addington J, et al. Individualized Prediction of Transition to Psychosis in 1,676 Individuals at Clinical High Risk: Development and Validation of a Multivariable Prediction Model Based on Individual Patient Data Meta-Analysis. Front Psychiatry. 2019;10:345.
25. Mechelli A, Lin A, Wood S, McGorry P, Amminger P, Tognin S, et al. Using clinical information to make individualized prognostic predictions in people at ultra high risk for psychosis. Schizophr Res. 2017;184:32-8.
26. Michel C, Ruhrmann S, Schimmelmann BG, Klosterkötter J, Schultze-Lutter F. A stratified model for psychosis prediction in clinical practice. Schizophr Bull. 2014;40(6):1533-42.
27. Nelson B, Yung AR. Can clinicians predict psychosis in an ultra high risk group? Aust N Z J Psychiatry. 2010;44(7):625-30.
28. Padmanabhan JL, Shah JL, Tandon N, Keshavan MS. The "polyenviromic risk score": Aggregating environmental risk factors predicts conversion to psychosis in familial high-risk subjects. Schizophr Res. 2017;181:17-22.
29. Paetzold I, Hermans K, Schick A, Nelson B, Velthorst E, Schirmbeck F, et al. Momentary Manifestations of Negative Symptoms as Predictors of Clinical Outcomes in People at High Risk for Psychosis: Experience Sampling Study. JMIR Ment Health. 2021;8(11):e30309.
30. Pawełczyk A, Łojek E, Żurner N, Kotlicka-Antczak M, Pawełczyk T. Higher order language impairments can predict the transition of ultrahigh risk state to psychosis-An empirical study. Early Interv Psychiatry. 2021;15(2):314-27.
31. Polari A, Yuen HP, Amminger P, Berger G, Chen E, deHaan L, et al. Prediction of clinical outcomes beyond psychosis in the ultra-high risk for psychosis population. Early Interv Psychiatry. 2021;15(3):642-51.
32. Riecher-Rössler A, Pflueger MO, Aston J, Borgwardt SJ, Brewer WJ, Gschwandtner U, et al. Efficacy of using cognitive status in predicting psychosis: a 7-year follow-up. Biol Psychiatry. 2009;66(11):1023-30.
33. Ruhrmann S, Schultze-Lutter F, Salokangas RK, Heinimaa M, Linszen D, Dingemans P, et al. Prediction of psychosis in adolescents and young adults at high risk: results from the prospective European prediction of psychosis study. Arch Gen Psychiatry. 2010;67(3):241-51.

34. Seidman LJ, Shapiro DI, Stone WS, Woodberry KA, Ronzio A, Cornblatt BA, et al. Association of Neurocognition With Transition to Psychosis: Baseline Functioning in the Second Phase of the North American Prodrome Longitudinal Study. JAMA Psychiatry. 2016;73(12):1239-48.
35. Studerus E, Beck K, Fusar-Poli P, Riecher-Rössler A. Development and Validation of a Dynamic Risk Prediction Model to Forecast Psychosis Onset in Patients at Clinical High Risk. Schizophr Bull. 2020;46(2):252-60.
36. Tarbox SI, Addington J, Cadenhead KS, Cannon TD, Cornblatt BA, Perkins DO, et al. Functional development in clinical high risk youth: prediction of schizophrenia versus other psychotic disorders. Psychiatry Res. 2014;215(1):52-60.
37. Thompson A, Nelson B, Yung A. Predictive validity of clinical variables in the "at risk" for psychosis population: international comparison with results from the North American Prodrome Longitudinal Study. Schizophr Res. 2011;126(1-3):51-7.
38. Uttinger M, Studerus E, Ittig S, Heitz U, Schultze-Lutter F, Riecher-Rössler A. The Frankfurt Complaint Questionnaire for self-assessment of basic symptoms in the early detection of psychosis-Factor structure, reliability, and predictive validity. Int J Methods Psychiatr Res. 2018;27(2):e1600.
39. Valmaggia LR, Stahl D, Yung AR, Nelson B, Fusar-Poli P, McGorry PD, et al. Negative psychotic symptoms and impaired role functioning predict transition outcomes in the at-risk mental state: a latent class cluster analysis study. Psychol Med. 2013;43(11):2311-25.
40. Worthington MA, Walker EF, Addington J, Bearden CE, Cadenhead KS, Cornblatt BA, et al. Incorporating cortisol into the NAPLS2 individualized risk calculator for prediction of psychosis. Schizophr Res. 2021;227:95-100.
41. Yuen HP, Mackinnon A, Nelson B. Dynamic prediction systems of transition to psychosis using joint modelling: extensions to the base system. Schizophr Res. 2020;216:207-12.
42. Yuen HP, Mackinnon A, Hartmann J, Amminger GP, Markulev C, Lavoie S, et al. Dynamic prediction of transition to psychosis using joint modelling. Schizophr Res. 2018;202:333-40.
43. Yung AR, Phillips LJ, Yuen HP, Francey SM, McFarlane CA, Hallgren M, et al. Psychosis prediction: 12-month follow up of a high-risk ("prodromal") group. Schizophrenia research. 2003;60(1):21-32.
44. The EPOS prediction model improves ability to predict transition to first episode psychosis in individuals at high risk. Evidence Based Mental Health. 2010;13(3):77-.
45. Zhang T, Xu L, Li H, Woodberry KA, Kline ER, Jiang J, et al. Calculating individualized risk components using a mobile app-based risk calculator for clinical high risk of psychosis: findings from ShangHai At Risk for Psychosis (SHARP) program. Psychological Medicine. 2021;51(4):653-60.
46. Zhang T, Xu L, Tang Y, Li H, Tang X, Cui H, et al. Prediction of psychosis in prodrome: development and validation of a simple, personalized risk calculator. Psychol Med. 2019;49(12):1990-8.
47. Zhang T, Yang S, Xu L, Tang X, Wei Y, Cui H, et al. Poor functional recovery is better predicted than conversion in studies of outcomes of clinical high risk of psychosis: insight from SHARP. Psychol Med. 2020;50(9):1578-84.
48. Zhang T, Li H, Tang Y, Niznikiewicz MA, Shenton ME, Keshavan MS, et al. Validating the Predictive Accuracy of the NAPLS-2 Psychosis Risk Calculator in a Clinical High-Risk Sample From the SHARP (Shanghai At Risk for Psychosis) Program. Am J Psychiatry. 2018;175(9):906-8.

Supplementary Material Table S3: PROBAST overview

Study Author	Participant Selection	Predictor	Outcome	Analysis	Overall
Addington (1)	+	+	-	-	-
Bang (2)	+	-	-	?	-
Bedi (3)	+	-	?	?	
Bourgin (4)	+	+	-	-	-
Brodey (5)	+	-	?	?	-
Cannon (6)	+	+	+	+	+
Cannon (7)	+	?	?	-	-
Carrion (8)	+	+	+	?	?
Chan (9)	+	-	?	-	-
Ciarleglio (10)	+	?	?	?	-
Conrad (11)	-	-	-	-	-
Dragt (12)	+	+	?	-	-
Formica (13)	+	?	+	-	?
Francesconi (14)	+	+	-	-	-
Fusar-Poli (15)	+	+	+	+	+
Guo (16)	+	+	?	-	-
Haidl (17)	+	-	?	-	-
Hengartner (18)	+	-	-	-	-
Ising (19)	+	+	+	-	-
Kotlicka-Antczak (20)	+	+	+	+	+
Koutsouleris (21)	+	?	+	-	-
Lee (22)	+	-	?	-	-
Lindgren (23)	+	?	-	-	-
Malda (24)	+	+	+	+	+
Mechelli (25)	+	-	-	?	-
Michel (26)	+	+	-	-	-
Nelson (27)	+	?	+	-	-
Padmanabha (28)	+	-	?	?	-
Paetzold (29)	+	?	+	-	-
Pawelczyk (30)	-	-	+	?	-
Polari (31)	+	-	?	-	-
Riecher-Rossler (32)	+	+	?	-	-
Ruhrmann (33)	+	+	-	+	?
Seidman (34)	+	-	?	?	-
Studerus (35)	+	+	?	+	-
Tarbox (36)	+	+	?	-	-
Thompson (37)	+	+	?	-	-
Uttinger (38)	+	-	-	?	-
Valmaggia (39)	-	?	-	-	-
Worthington (40)	+	-	-	-	-
Yuen (41)	?	-	-	-	-
Yuen (42)	-	+	-	-	-

Yung (43)	+	+	?	-	-
Yung (44)	+	+	?	?	?
Zhang (45)	+	-	?	?	-
Zhang (46)	+	-	?	-	-
Zhang (47)	+	?	?	-	-
Zhang (48)	?	+	?	-	-
High ROB (+)	4	17	15	31	40
Low ROB (-)	42	22	11	6	4
Unclear ROB (?)	2	9	22	11	4

Supplementary Material Table S4: Frequency of Predictors

Predictors	Definition	Studies reporting predictor (N)
**GAF score**	Global Assessment of Functioning – the assessment of the effect of psychological symptoms on an individual's daily life	17
**Age**	Age of participant at time of completion	14
**Trait group**	Genetic vulnerability	11
**UTC**	Unusual thought content assessed via SIPS	9
**Gender**	Sex assigned at birth	8
**Positive symptoms**	Assessed via SIPS, referring to an excess of vital properties, such as clonic jerking, abnormal movement, hallucinations and delusions.	8
**SOFAS score**	Social and occupation functioning assessment scale	7
**Decline in GAF**	An assessment of the decline in Global Assessment of Functioning.	7
**Sums of SIPS**	The sum of Unusual Thought Content (SIPS P1) and Suspiciousness (SIPS P2)	7
**BACS**	Brief Assessment of Cognition in Schizophrenia	6
**HVLT-R**	Hopkins Verbal Learning Test	6
**APS**	Attenuate psychosis syndrome (frequency and duration)	5
**CAARMS**	CAARMS assessment	5
**SCID**	Substance abuse	5
**Social anhedonia**	Ability to experience pleasure in a social environment	5
**Ethnicity**	The ethnicity of individual	4
**Years of education**	Individuals' years of formal education	4
**Sleep disturbances**	Individuals' ability to sleep throughout	3
**Suspicion/paranoia**	Assessed via BPRS	3
**Suspicion/paranoia**	Assessed via SIPS	3
**Ideational richness**		3
**Anhedonia**	Ability to experience pleasure in any/all settings	3
**Trauma**		3
**Executive and visuospatial abilities**		3
**DUS**	Duration of untreated symptoms	3
**Verbal memory**		3
**IQ**		2
**Occupation**		2
**Disorganised speech**	Assessed via CAARMS	2
**Duration between symptom onset and treatment**		2
**BLIPS**	Brief Limited Intermittent Psychotic Symptoms (BLIPS) – defined individuals at ultra-high risk for psychosis	2
**Organisation/cognitive**		2

**Negative symptoms sum**	Assessed via SIPS and refers to the negation of vital properties, resulting in loss of sensation, paralysis, and coma.	2
**History of sexual trauma**		2
**Avolition**	The decrease in the ability to initiate and persist in self-directed purposeful activities.	2
**Expression of emotion**		2
**Stressful life events**		2
**Attention and working memory abilities**		2
**Depression**	Assessment of depression among individual	2
**Verbal fluency**		2
**Urban upbringing**		2
**SFS**	Social engagement/withdrawal in Social Functioning Scale	1
**Strange stories task**	Results of a strange stories task	1
**CVLT**	California Verbal Learning Test	1
**Medication prescription**		1
**Anxiety**		1
**Age by sex interaction**		1
**Migrant status**		1
**Winter or spring birth**		1
**Marital status**		1
**Disorganisation symptoms**	Assessed via SIPS	1
**General Symptoms**	Assessed via SIPS	1
**Transition risk**		1
**MADRS**	Montgomery Asberg Depression Rating Scale	1
**YMRS**	Young Mania Rating Scale	1
**Recruiting site**	Location of recruiting site	1
**PBIQ-R**	Personal Beliefs about Illess Questionnaire	1
**Treated by PAS**		1
**Suicidal behaviours**		1
**Suicidal ideation**		1
**Violent behaviours**		1
**Violent ideation**		1
**Trouble with focus and attention**		1
**Perplexity and delusional mood**		1
**Motor disturbances**		1
**Experience of emotions and self**		1
**Impairment in personal hygiene**		1
**Trial making test**		1
**BVMT**	Brief Visuospatial Memory Test	1
**Functioning decline**		1

**Dysphoric mood**	The mental state of an individual whereby the individual has a profound sense of unease or dissatisfaction	1
**Aggression/dangerous behaviours**		1
**Mood swings/liability**		1
**OCD**	Obsessive compulsive disorder	1
**DSRS**	Digital symbol raw score	1
**Physical abuse**		1
**History of neglect**		1
**Parental death**	History of the death of a parent	1
**Advanced paternal age**	Pregnant individuals greater than 40-45 years old at time of delivery	1
**Obstetric and perinatal complication**	Complications during birth, such as delivery by caesarean section, preterm birth, low birthweight	1
**LEE irritability score**	Four factor questionnaire measuring the expressed emotions (EE) as perceived by the individual	1
**ROFT**	Visual memory	1
**WCST, Wisconsin card scoring test**	Set shifting and problem solving score	1
**Lexical and Semantic Category Tasks**	Assessment of verbal executive functions	1
**Processing speed**	Assessed via DST and TMT	1
**Positive symptoms**	Assessed via BPRS-E	1
**Disorganisation**	Assessed via BPRS-E	1
**NES**	Sensory integration	1
**FPT**	Faux pas test	1
**Suicidal attempt**	An attempt of taking own life	1
**Police contact**	History of police contact with individual	1
**Academic maladjustment**		1
**Personality disorder**	History/existence of a personality disorder for individual	1
**Physical disorder**	History/existence of a physical disorder for individual	1
**Other mental health problems**	History/existence of other known mental health disorders	1
**Number of therapy sessions**	Number of therapy session; Including supportive theory, problem solving, risk management, motivational interviewing, psychoanalysis, CBT.	1
**CBT Sessions**	History of Cognitive behavioural therapy (yes/no)	1
**Problem solving**	Assessment	1
**Study cohort**	Year of cohort	1
**Sematic coherence**	Automated test analysis	1
**PAS Items**	Premorbid social adjustments	1
**Receiving state benefit**	Yes/no	1
**Syntactic assays**	Automated test analysis	1

Supplementary Material Figure S5: Events Per Variable

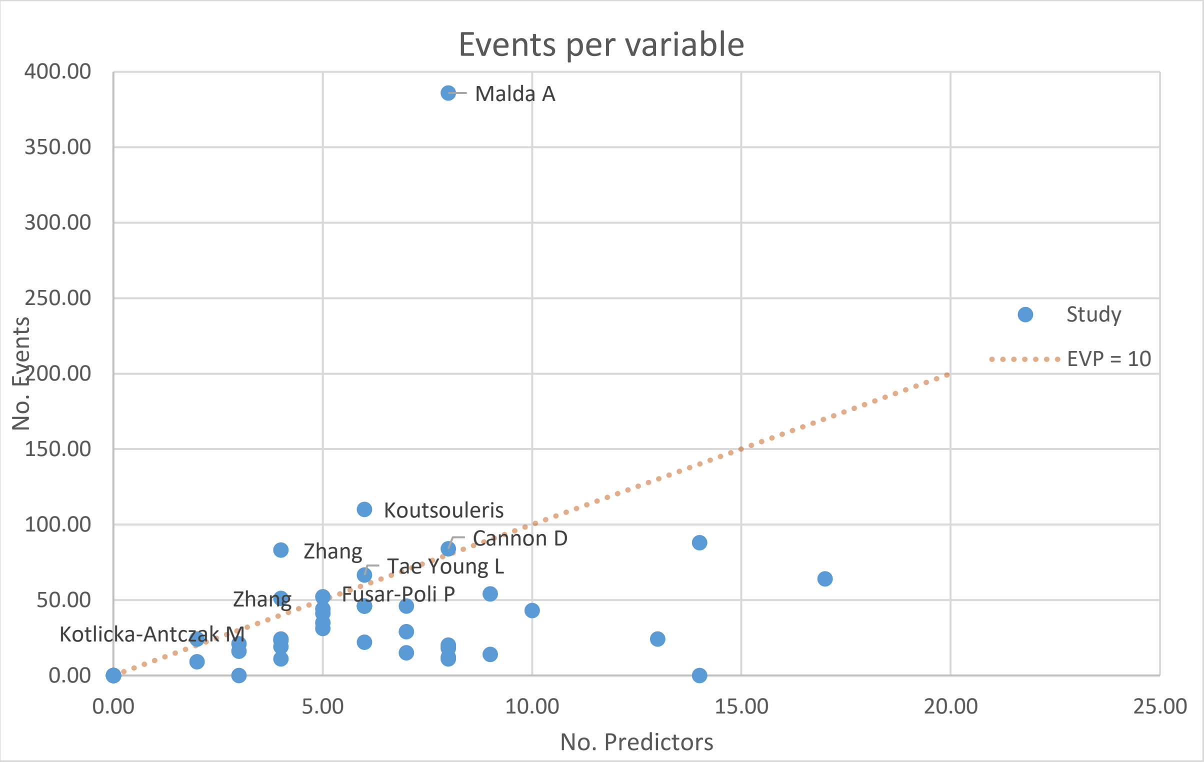

Supplementary Table S6: Predictors from the 4 studies identified as low risk of bias overall

Model	Cannon (6)	Fusar-Poli (15)	Kotlicka-Antczak (20)	Malda (24)
**Predictors included:**				
Age	x	x	-	x
APS	-	-	-	x
BLIPS	-	-	-	x
Digital symbol (raw)	x	-	-	-
GAF	x	-	-	-
Genetic risk	x	-	-	x
HVLT	x	-	-	-
Index diagnosis	-	x	-	-
Negative symptoms	-	-	-	x
Positive symptoms	-	-	-	x
Race/ethnicity	-	x	-	-
Sex	-	x	-	x
Speech disorganisation	-	-	x	-
Stressful life events	x	-	-	-
Sum of SIPS	x	-	-	x
Traumas	x	-	-	-
Unusual thought content	-	-	x	-

Supplementary Table S7: Performance statistics for identified studies graded as low risk of bias

Model	Calibration Slope *	Apparent discrimination†	Bootstrap-adjusted discrimination‡
**Cannon (6)**			
Model for use Development model (β terms)			0.71
**Fusar-Poli (15)**			
Model for use Development model (β terms)		0.80 [0.785-0.816]	
**Kotlicka-Antczak (20)**			
Model for use Development model (β terms)		0.79	0.78
**Malda (24)**			
Model for use Development model (β terms)	0.886 [0.745-1.022]	0.655 [0.627-0.682]	

*Bootstrap Calibration slope
†C-statistic based on development data
‡C-statistic based on bootstrap internal validation
